# Primary Transitional Cell Carcinoma of the Endometrium: A Case Report and Review of Literature

**DOI:** 10.7759/cureus.21365

**Published:** 2022-01-18

**Authors:** Razan Amjad, Hussain Mogharbel

**Affiliations:** 1 Radiation Oncology, University of Manitoba, Winnipeg, CAN; 2 Radiation Oncology, King Abdulaziz University, Rabigh, SAU; 3 Obstetrics and Gynecology, University of Manitoba, Winnipeg, CAN; 4 Obstetrics and Gynecology, King Abdulaziz University, Jeddah, SAU

**Keywords:** transtional cell, endometrium, cytokeratin 20, cytokeratin 7, transitional cell carcinoma of the endometrium

## Abstract

Transitional cell carcinoma of the endometrium is a rare cancer. We present a 58-year-old Caucasian female was diagnosed with high-grade polyploid transitional cell carcinoma of the endometrium. The pathological findings included a dense serosal adhesion over the fundus and the posterior wall, necrotic and polyploid papillary mass in the endometrial cavity, serosal adhesions and unremarkable parenchyma in the fallopian tubes. Others were the presence of cancer antigen 125 and cytokeratin 7 and absence of cytokeratin 20, actin, estrogen receptor, soluble protein 100, vimentin, and cytokeratin high molecular weight.

The case is relevant to practice as it identifies the histological patterns for primary transitional cell carcinoma of the endometrium, including expressing cytokeratin 7 instead of cytokeratin 20. The tumor showed a papillary and solid architecture and composed of undifferentiated to poorly differentiated cells with no defined glandular nor squamous differentiation.

## Introduction

Transitional cell carcinoma of the endometrium (TCCe) is a rare condition as only a few cases have been reported. Consequently, the study describes a unique case of primary TCCe with 100% transitional cell differentiation and reviews past studies on the subject. Researchers have examined primary TCCe including the histological characteristics and diagnostic criteria. However, there are concerns regarding the histological patterns and diagnosis of the condition as no specific criteria have been developed. Therefore, the study presents a case report on primary TCCe and discusses the patient’s pathological findings based on past studies to add to the knowledge and body of literature.

## Case presentation

**Figure 1:**
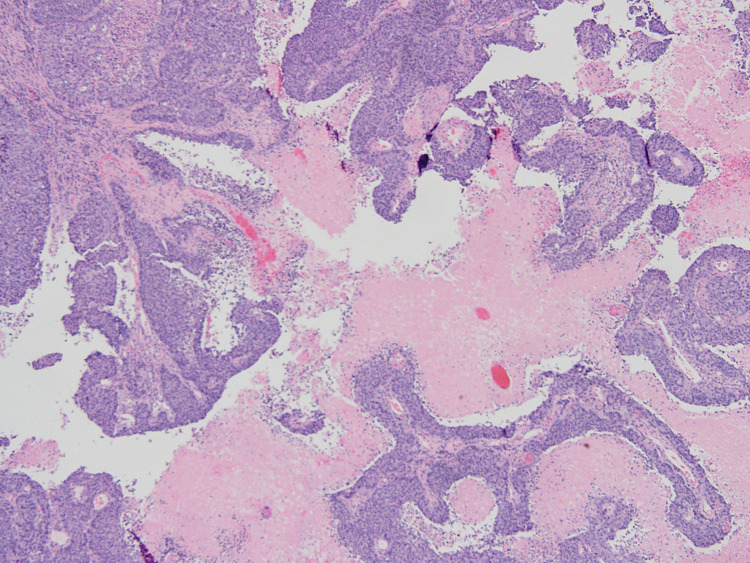
Low-power view of the tumor showing papillary and solid architectural patterns with fine fibrovascular cores and multilayered epithelial cells (transitional epithelium). (Hematoxylin and eosin x40 magnification).

**Figure 2:**
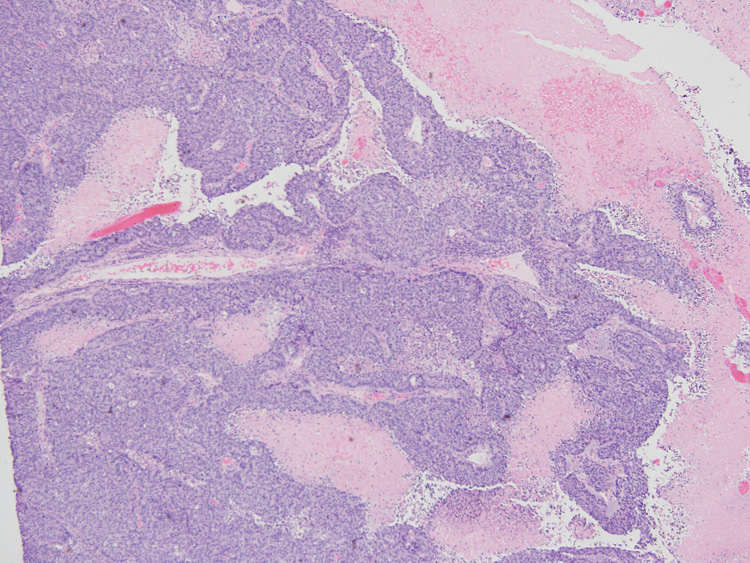
Low-power view of the tumor showing papillary and solid architectural patterns with fine fibrovascular cores and multilayered epithelial cells (transitional epithelium). (Hematoxylin and eosin x40 magnification)

**Figure 3:**
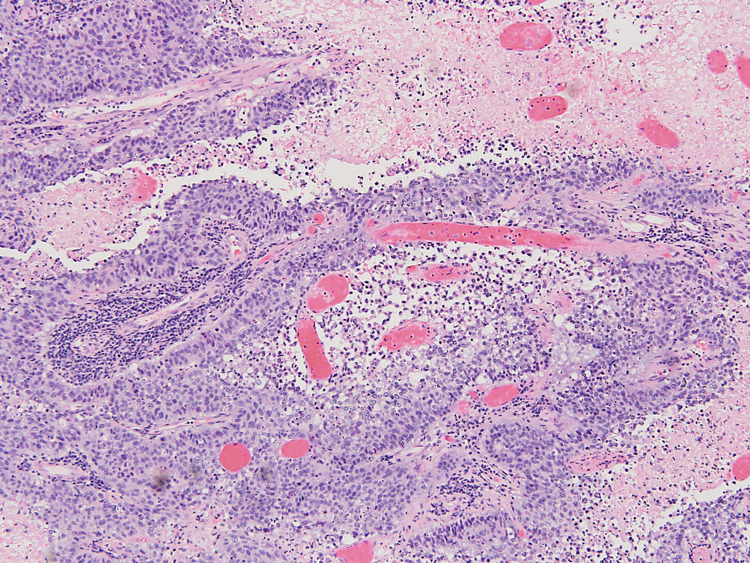
High-power view of the tumor showing papillary fronds with transitional epithelium, mixed inflammatory infiltrate, and necrosis. (Hematoxylin and eosin x200 magnification). The tumor cells have round to oval pleomorphic nuclei with vesicular to coarsely clumped chromatin, inconspicuous nucleoli, abundant eosinophilic cytoplasm, and indistinct cell borders.

A 58-year-old Caucasian woman who had given birth to three children presented to the general practitioner with postmenopausal bleeding for 20 days. She had her menopause at age of 53 years. Initial pelvic ultrasound showed thickened endometrium at 8 mm, no other gross pathology. An endometrial biopsy was done, and it indicated the patient had a poorly differentiated adenocarcinoma of the endometrium. Initial pelvic ultrasound showed thickened endometrium at 8 mm and no other gross pathology. The patient underwent a total abdominal hysterectomy, washings, and bilateral salpingo-oophorectomy with bilateral pelvic lymph node dissection. Adjuvant treatment and radiotherapy were not initiated. Cystoscopy revealed the patient had a normal bladder and she has been well five years after surgery. The patient's specimens were placed in formalin and the uterus measured 11.5 x 7.5 x 6.5 cm. The examination showed dense serosal adhesion over the posterior wall and fundus. In addition, the endometrial cavity was cavernous and had a big necrotic polyploid papillary mass that was connected to the fundus at the right cornu by a pedicle that stretched 6 cm of the internal OS. The remainder of the uterus, tubes, ovaries, and cervix was normal. The examination of the pelvic lymph nodes showed no signs of metastasis and the abdominal washings had no malignancy. Immunohistochemistry confirmed the presence of cancer antigen 125 (CA125) and cytokeratin 7 (CK7) and absence of cytokeratin 20 (CK20), actin, estrogen receptor (ER), soluble protein 100 (S100), vimentin, and cytokeratin high molecular weight (CK HMW). Tumor protein P53 (P53) staining was positive, diffuse, and strong. The tumor showed a papillary and solid architecture, and the cells were undifferentiated or poorly differentiated with no clear glandular and squamous differentiation (Figures 1-3) [1,2].

We found that transitional cell carcinomas affect few people, and only a limited number has been reported in studies. However, the analysis of the 58-year-old patient provided more information on the histological features of primary TCC that can be included in the diagnostic criteria. According to the pathological findings and the review of literature, the tumor should express CK7 rather than CK20 [1-3]. Moreover, the tumor should have papillary and the cells are differentiated, undifferentiated, or unsuccessfully differentiated. The endometrial carcinoma should be grouped as transitional cell carcinoma if 50% of the whole tumor is a transitional cell type.

## Discussion

Transition cell carcinoma (TCC) is a rare neoplasm to find in a woman's reproductive tract. TCC of the ovary is more prevalent than endometrium, cervix, and fallopian tube TCC. Therefore, only 21 cases of TCCe and endometrial carcinoma with TCC differentiation (TCCD) have been identified in past studies. The component volume of transitional cell differentiation in cases documented ranges between 5% and 95% of the tumors, details of all reported cases are provided in (Table 1) [1-9]. A range of aspects concerning TCCe is controversial such as histogenesis and the function of human papillomavirus (HPV) as a cause. Endometrial transitional cell carcinoma is prevalent in females who have reached menopause and causes postmenopausal spotting in most of the cases.

**Table 1:** Pathological findings show transitional cell carcinoma in the endometrium and endometrial carcinomas with transitional cell deafferentation. NA: not applicable; NED: no evidence of disease; TCCe: transitional cell carcinoma of the endometrium

Source	Case	Age (years)	Size (cm)	Associated component	Transitional cell carcinoma component	Myometrial invasion	Figo stage	Follow-up
This case	1	58	0.1	Squamous	100%	NA	II	Alive
Mariño-Enríquez et al. [4]	2	63	3.7	Endometroid (G2)	50%	Inner half (6 mm)	IB	Alive, NED at 16 months
	3	77	5.5	Endometroid (G3)	90%	Inner half (3 mm)	IIB	Alive, NED at 8 months
	4	55	4	None	100%	Inner half (6 mm)	IB	Alive, NED at 6 months
	5	73	6.5	Endometroid (G1)	20%	NA	IB	Alive, NED at 6 months
	6	76	NA	Endometroid (G2)	80%	Inner half (5 mm).	IB	Alive, NED at 3 months
Chen [6]	7	71	5.5	Mucinous glandular	95%	Outer half	IIIA	Alive, NED at 5 years
Spiegel et al. [5]	8	75	4	Endometroid (G1)	75%	Inner third	IIIA	Alive, NED at 15 months
Lininger et al. [3]	9	41	0.8	Endometrpid and papillary	40%	32%	IIIA	Follow-up: lost
	10	43	2	Endometroid and squamous	80%	67%	IC	Follow-up: lost
	11	53	3	Endometroid and mucinous	5%	29%	IB	Alive, NED at 4 months
	12	60	1.5	Squamous	95%	NA	I	Follow-up: lost
	13	64	7	Endometroid, papillary and serous	70%	80%	IC	Alive, NED at 35 months
	14	73	0.4	Squamous	95%	NA	I	Dead due to colon cancer with metastasis at 13 years of follow-up
	15	76	5	Endometroid and squamous	75%	95%	IC	Recurrence at 12 months
	16	83	3.2	Squamous and spindle cell sarcoma	50%	38%	IB	Alive, NED at 8year
Fukunaga and Ushigome [2]	17	50	10	Endometroid	95%	Inner third	IB	Alive, NED at 7 year
Labonte et al. [7]	18	46	8	Endometroid	90%	Outer half	IV	Alive, NED at 18 months
Lum [8]	19	77	8.6	NA	NA	Pelvic organs	IV	Alive, NED at 18 months
Ahluwalia et al. [1]	20	78	2	Endometroid (G3)	N95%	1mm	IB	Alive, NED at 10 months
Giordano et al. [9]	21	61	1.5	None	100%	Not invasion IB	IB	Not applicable
Ribeiro-Silva [10]	22	84	5	Papillary squamous	15%	Inner half	IB	Died after 1 year because of TCCe

In the current case, the female patient presented complained of postmenopausal bleeding. A range of reports has associated the morphological changes with the aggressiveness of the tumor and response to radiation treatment. Tumors are considered TCCe when more than 50% of the mass pathology is consistent with TCC. Lesions that have no more than 50% of the transitional cell carcinoma are deemed as endometrial carcinoma with TCCD. The differentiation of the TCCe is a neometaplastic change from endometrioid carcinoma. Transitional cell carcinoma of the endometrium has CK7 instead of CK20 while urinary bladder papillary transitional cell carcinoma contains the two. Tumors in the genital tract show CK7 and not CK 20, whereas mucinous tumors in the ovary express CK20 and CK7. The CK7 and CK20 were present in five of the seven cases of TCCe evaluated by Lininger et al., they compared them with nine cases of transitional cell carcinoma of the urinary bladder that showed eight cases had CK7 instead of CK20 and one had no CK7, but not CK20 [1-3]. Similarly, Mariño-Enríquez et al. in their study found that in five patients with TCC, the transitional carcinoma tested positive for CK7 and not CK20 [4]. The immunohistochemistry findings for the 58-year-old patient were in line with results from previous studies. The TCC was positive for CK7 and negative for CK20. Therefore, the TCCe like the TCCs of the cervix has a Mullerian source contrary to urothelial TCC. Endometrial TCC has morphological similarities to urothelial papillary but varies in the immunophenotypic characteristics and represents different types of tumors. Researchers have identified histological characteristics of primary TCCe to facilitate the diagnosis of the differential condition. Lininger et al. noted eight cases of primary TCCe had different histological features, for instance, endometroid, sarcomatous, serous, and unsuccessfully differentiated cells [3]. Additional characteristics included squamous, papillary, and villo-glandular differentiation. Five out of the seven cases of TCCe had CK7 and the intensity ranged between 20% and 100%. However, they did not demonstrate immunoreactivity for CK20. Table 2 provides a summary of the immunohistochemical results for the nine cases.

**Table 2:** Immunohistochemical staining results for cytokeratin 7 and cytokeratin 20 in transitional cell carcinoma of the endometrium. Immunohistochemical staining was done to determine the presence of cytokeratin 7 (CK7) and cytokeratin 20 (CK20) in the transitional cell carcinomas of the endometrium. The staining intensity was determined and expressed as a percentage of the transitional cell carcinoma component that showed immunoreactivity and graded from 0 to 3. Source: Courtesy to Lininger et al. (permission to use data was obtained) [3].

Case number	Intensity of staining (%)		Cytokeratin 7 or cytokeratin 20 profile
	Cytokeratin 7	Cytokeratin 720	
1	2-3 (100%)	0 (0%)	CK7/CK200
2	NA	NA	NA
3	3 (100%)	0 (0%)	CK7/CK200
4	0 (0%)	0 (0%)	CK7/CK200
5	3 (100%)	1 (5%)	CK7/CK200
6	0 (0%)	0 (0%)	CK70/CK200
7	2 (20%)	0 (0%)	CK7/CK200
8	1-2 (70%)	0 (0%)	CK7/CK200
9	N/A	N/A	N/A

In addition, Fukunaga and Ushigome in their study found that the patient had endometrial lesion that was histologically similar to TCC grade 2 or 3 though it differed from endometrial carcinomas. The focal region was less than 5% of endometrioid carcinoma lesion and the change between the urothelial epithelium and the glandular and squamous differentiation [2].

The differentiation of endometroid tumors and TCC is a concern because of the lack of diagnostic criteria. The endometrial tumors should not be diagnosed based on squamous differentiation and papillary structures that are covered by thick epithelium according to Ahluwalia et al. [1]. A morphological criterion that can be used to diagnose transitional cell components in endometrial carcinoma, which show a connection with TCC particularly grade 2 and 3 of the urinary bladder and urinary tract should be identified. Differential diagnosis of TCCe should entail poorly differentiated endometroid carcinoma and metastatic transitional cell carcinoma of the ovary and urinary tract. Direct extension of the primary TCC to the cervix should be eliminated. Transitional cell carcinoma is differentiated from poorly differentiated endometrial adenocarcinoma by the papillary and solid tissue pattern similar to the TCC of the urinary tract system [5].

Endometrial carcinoma is categorized into various grades, including high grade, low grade, grade 1, 2, and 3 [2]. The pathological findings depend on the grade at the time of diagnosis, including differentiated, incompletely differentiated, and undifferentiated cells. Grade 3 endometrial adenocarcinoma has squamous components, though focal squamous differentiation is also common in TCCe. The diagnosis will depend on whether the tumor is similar to the urinary tract TCC.

In a study by Spiegel et al., a biopsy was done and the patient was diagnosed with grade 3 endometrial carcinoma because of the big component of the solid and papillary regions of unidentified transitional cell carcinoma [5]. The pathology findings from the present case study showed the patient had a high-grade endometrial carcinoma as the tumor consisted of undifferentiated and poorly differentiated cancer cells, but it did not have a specific glandular and squamous differentiation. Additionally, the tumor also showed a solid architecture and a papillary [2-4].

## Conclusions

In conclusion, transitional cell carcinomas affect few people, and only a limited number has been reported in studies. However, the analysis of the 58-year-old patient provided more information on the histological features of primary TCC that can be included in the diagnostic criteria. According to the pathological findings in our study and review, the tumor should express CK7 rather than CK20. Moreover, the tumor should have a papillary and the cells are differentiated, poorly differentiated, or undifferentiated. The endometrial carcinoma should be grouped as transitional cell carcinoma if 50% of the whole tumor is a transitional cell type.
